# Targeting transglutaminase 2: pathways to celiac disease therapies

**DOI:** 10.1093/gastro/goaf086

**Published:** 2025-10-03

**Authors:** Alexandra Endrizzi, Pauline Grunst, Silvia Rudloff, Jan De Laffolie, Klaus-Peter Zimmer, Sebastian Stricker

**Affiliations:** Department of General Pediatrics and Neonatology, Justus Liebig University Giessen, Giessen, Germany; Department of General Pediatrics and Neonatology, Justus Liebig University Giessen, Giessen, Germany; Department of General Pediatrics and Neonatology, Justus Liebig University Giessen, Giessen, Germany; Department of Nutritional Science, Justus Liebig University Giessen, Giessen, Germany; Department of General Pediatrics and Neonatology, Justus Liebig University Giessen, Giessen, Germany; Department of General Pediatrics and Neonatology, Justus Liebig University Giessen, Giessen, Germany; Department of General Pediatrics and Neonatology, Justus Liebig University Giessen, Giessen, Germany

**Keywords:** celiac disease, transglutaminase 2, siRNA, TG2 inhibition, TRX, ERP57

## Abstract

**Background:**

Transglutaminase 2 (TG2)-mediated enzymatic modification of gliadin peptides plays a major role in the pathogenesis of celiac disease (CD). Different inhibitory mechanisms have been reported to reduce TG2 activity but comparative data on the cellular level are lacking. Furthermore, recent evidence suggested that endogenous redox proteins such as endoplasmic reticulum resident protein 57 (ERp57, inhibits TG2) and thioredoxin-1 (TRX, activates TG2) may regulate TG2 activity. In this study, we aimed to compare the effects and applicability of different inhibitors on the activity of recombinant and cellular TG2. Furthermore, we investigated the role of ERp57 and TRX in the context of CD by using siRNA-mediated knockdown in Caco-2 cells.

**Methods:**

The effect of TG2 inhibitors on recombinant and extracellular TG2 activity was investigated by using photometric and fluorometric quantitation of the cross-linking of biotinylated gliadin peptide P56-88 or 5-(biotinamido)-pentylamine. After siRNA knockdown, the protein levels of ERp57, TRX, and TG2 as well as TG2 activity were investigated by using Western blotting and fluorometry in Caco-2 cells.

**Results:**

The active-site-directed inhibitors ERW1041, KCC009, and cysteamine as well as the allosteric inhibitor LDN27219 revealed the most prominent reduction in recombinant and cellular (35%–50%) TG2 activity. In contrast, PX12, *S*-Nitroso-*N*-acetyl-DL-penicillamine, zinc chloride, and ascorbic acid either did not affect TG2 activity or had only moderate effects at high doses close to cytotoxic concentrations. SiRNA knockdown of TG2 resulted in a prominent reduction (63%) in TG2 activity, whereas knockdown of ERp57 did not; knockdown of TRX only slightly (27%) reduced TG2 activity.

**Conclusion:**

Active-site-directed inhibitors, LDN27219 and knockdown of TG2 expression significantly reduced extracellular TG2 activity and represent potential alternative treatment targets in the context of CD.

## Introduction

Celiac disease (CD) is a frequent autoimmune disease (1% of the Western civilization) triggered by the ingestion of gliadin and related prolamines in genetically predisposed (HLA-DQ2/DQ8 genotype) individuals. Transglutaminase 2 (TG2) plays an essential role in the pathogenesis of CD, as it specifically modifies distinct gliadin peptides by transamidation and deamidation [1]. Deamidation of α-gliadin increases its affinity towards the HLA-DQ2 receptor and promotes antigen presentation. The following Th1-mediated immune response results in the production of cytokines such as interferon-γ (IFN-γ) and tumor necrosis factor-α (TNF-α) and the destruction of the intestinal mucosa [2–5]. Transamidation and/or cross-linking of gliadin peptides are thought to induce the production of autoantibodies including anti-TG2-IgA antibodies and to promote secondary autoimmunity [6, 7].

Apart from its pathogenetic role in CD, TG2 has multiple physiological functions in the intra- and extracellular compartment, e.g. the modulation of autophagy and cell cycle as well as tissue repair, which require strict regulation of its enzymatic activity [8]. In the intracellular compartment, TG2 resides in an inactive state (closed conformation), promoted by the guanosine nucleotides GDP/GTP and a low calcium concentration. In the extracellular compartment, the presence of allosteric disulfide bonds (cysteine residues 370–371) under oxidizing conditions contributes to the inactive state of TG2 [9, 10]. As extracellular TG2 plays such a detrimental role in the pathophysiology of CD, it serves as a target for potential pharmacological treatment. Unselective, competitive amine donors (e.g. cysteamine) and TG2-specific substances (e.g. ERW1041, KCC009) that irreversibly bind to the active center have been widely used in cell culture and murine models [11–15]. Recently, the TG2 inhibitor ZED1227 underwent clinical evaluation in a Phase II trial and attenuated gluten-induced duodenal mucosal damage in CD patients [16, 17].

Besides active-site-directed inhibition, allosteric processes such as the oxidation of disulfide bonds by PX12, GTP antagonism by LDN27219, and *S*-nitrosylation of thiol groups by *S*-Nitroso-*N*-acetyl-DL-penicillamine (SNAP) have also been described [12, 18–21]. Lately, the calcium antagonism of zinc chloride and the complexation of gliadin with ascorbyl palmitate were also suggested as potential treatment targets [22, 23]. In addition to exogenous TG2 inhibitors, the influence of endogenous redox proteins on TG2 activity and their potential role in CD have received attention recently.

In this context, thioredoxin-1 (TRX) was shown to activate extracellular TG2 by reducing the specific disulfide bond in cell culture and murine models [24–26]. TRX is part of an essential cytosolic redox system, works as an antioxidant, and modulates inflammation and apoptosis [27, 28]. High expression of TRX was associated with multiple inflammatory conditions such as rheumatoid arthritis, acute pancreatitis, sepsis or bronchial asthma, and multiple tumor diseases [27, 29]. In addition to its intracellular functions, TRX is also secreted into the extracellular matrix by immune cells [24, 25, 30, 31].

In contrast, the oxidative protein endoplasmic reticulum resident protein 57 (ERp57, also known as protein disulfide isomerase A3) has been shown to inactivate extracellular TG2 by oxidation in a cell culture model [32]. ERp57 contributes to proper protein folding and the assembly of the major histocompatibility complex MHC-I [33].

However, the contribution of endogenous oxidative proteins to the regulation of cellular TG2 activity has hardly been addressed. Furthermore, an in-depth comparative investigation of the effect of different inhibitory mechanisms on TG2 activity is lacking to date.

We therefore performed the first study to compare the influence of active-site-directed and various allosteric inhibitors on the activity of recombinant and extracellular TG2. Furthermore, we investigated the effects of the siRNA-mediated knockdown of ERp57, TRX, and TG2 on TG2 activity in a cell culture model.

## Materials and methods

### Patient characteristics

All duodenal biopsies were obtained during clinically indicated gastrointestinal endoscopy. The study was conducted in accordance with the principles outlined in the Declaration of Helsinki and was approved by the local ethics committee (reference number 146/16); written informed consent was obtained for every patient. CD was diagnosed according to current guidelines of the European Society for Paediatric Gastroenterology, Hepatology and Nutrition [34]. Mucosal expression of ERp57, TRX, and TG2 was investigated by using indirect confocal microscopy in duodenal biopsies of CD patients and controls (Table 1).

**Table 1.** Patient characteristics

Patient	Group	Sex	Age	Anti-TG2-IgA (IU/ml)	Marsh grade	Remarks
**1**	Control	M	13	<2	0	Eosinophilic esophagitis
**2**	Control	F	15	<2	0	Gastritis type C
**3**	Control	F	14	<2	0	Reflux esophagitis
**4**	Control	M	5	<2	0	Reflux esophagitis
**5**	Control	F	15	<2	0	Functional abdominal pain
**6**	CD	M	7	>200	3a	
**7**	CD	M	5	>200	3c	
**8**	CD	F	10	90	3b	
**9**	CD	F	5	134	3a	
**10**	CD	M	3	>200	3a	Trisomy 21, atrioventricular septal defect

Cut-off anti-TG2-IgA > 20 IU/ml. M = male, F = female.

### *In vitro* transamidation of gliadin peptide P56-88 by recombinant TG2

The detailed assay principle has been published elsewhere [12]. Briefly, 96-well plates were coated with 10 nM of TG2 (Zedira, Darmstadt, Germany). Incubation with biotin-conjugated gliadin peptide P56-88 (LQLQPFPQPQLPYPQPQLPYPQPQLPYPQPQPF; Biosynth, Lelystad, The Netherlands) with or without the corresponding inhibitors was performed at 37°C for 1 h (Table 2). The cross-linked gliadin peptide was detected by using horseradish peroxidase (HRP)-conjugated streptavidin (2 µg/mL; Biolegend, San Diego, CA, USA) and visualized with the HRP-substrate tetramethylbenzidine (Sigma-Aldrich, Darmstadt, Germany). Photometric quantitation at 655 nm was performed after 10 min by using a Clariostar Plus microplate reader (BMG Labtech, Ortenberg, Germany).

**Table 2.** Inhibitors used

Substance	Group	Mechanism	Company
ERW1041	Active-site-directed inhibitors	Irreversible, TG2-specific binding to the cysteine residue in the active center	5095220001, Sigma-Aldrich, Darmstadt, Germany
KCC009			HY-123290, MedChemExpress, Monmouth Junction, NJ, USA
Cysteamine		Competitive amine inhibition	M6500, Sigma-Aldrich, Darmstadt, Germany
PX12	Allosteric inhibitors	Oxidation of allosteric disulfide bonds	M5324, Sigma-Aldrich, Darmstadt, Germany
LDN27219		GTP antagonism, stabilization of closed (inactive) conformation of TG2	HY-16693, MedChemExpress, Monmouth Junction, NJ, USA
*S*NAP		*S*-nitrosylation of thiol groups	HY-121526, MedChemExpress, Monmouth Junction, NJ, USA
Zinc chloride (ZnCl_2_)	Calcium antagonism	Competitive calcium antagonism	208086, Sigma-Aldrich, Darmstadt, Germany
Ascorbic acid (AA)	Gliadin complexation	Complexation of immunogenic gliadin peptides	A92902, Sigma-Aldrich, Darmstadt, Germany

### Cell culture

Human intestinal epithelial cells Caco-2 (DSMZ-German Collection of Microorganisms and Cell Cultures GmbH, Braunschweig, Germany) were cultured as previously described [12]. Stimulation with 100 or 1,000 IU/mL of IFN-γ (Sigma-Aldrich) was carried out for 48 h before the cells were harvested.

### Cell viability

The effect of the inhibitors on the viability of Caco-2 cells was investigated by using the resazurin-based PrestoBlue HS assay (Thermo Fisher Scientific, Langenselbold, Germany) as described elsewhere [12]. Fluorometric examination was conducted by using a Clariostar Plus microplate reader.

### siRNA transfection

The siRNA transfection was performed in Caco-2 cells at 60%–80% confluence. In brief, lipofectamine 2000 (1:100; 11668019; Thermo Fisher Scientific) and the corresponding siRNAs (1:50; mock siRNA, *ERp57*-siRNA, *TRX*-siRNA, and *TG2*-siRNA from Santa Cruz Biotechnology, Dallas, TX, USA) were diluted in Opti-MEM medium (Thermo Fisher Scientific). The diluted lipofectamine 2000 and the siRNA (1:1, v:v) were incubated for 20 min at room temperature and the mixture was added to the cells (total volume 96-well plate 50 µL, 24-well plate 100 µL). After 5 h of incubation, Caco-2 medium (96-well plate 150 µL, 24-well plate 500 µL) was added and the cells were further incubated until 48 h after transfection.

### Western blotting

The detailed Western blotting protocol using Caco-2 cell lysates has been published elsewhere [12]. Primary antibodies (Table 3) were incubated at 4°C overnight. The next day, the membrane was incubated with the corresponding HRP-conjugated secondary antibody (anti-rabbit-IgG from Cell Signaling Technology, Danvers, MA, USA; anti-mouse-IgG from Santa Cruz Biotechnology) for 1 h. The blotting membrane was developed by using the SuperSignal West Femto kit (Thermo Fisher Scientific) for 5 min. Image processing and quantitation were performed by using a ChemiDoc XRS+ imager and Image Lab software (BioRad Laboratories, Feldkirchen, Germany).

**Table 3.** Antibodies used

Target	Antibody	Host/clonality	Concentration	Company
ERp57	15967-1-AP	Polyclonal/rabbit	WB: 1:5,000 in 5% BM in PBST FL: 1:500 in 5% BSA CF: 1:200 in blocking solution	Proteintech, Planegg-Martinsried, Germany
GAPDH	MAB374	Monoclonal/mouse	WB: 1:5,000 in 5% BM in PBST	Merck Millipore, Darmstadt, Germany
TG2	D11A6	Monoclonal/rabbit	WB: 1:5,000 in 5% BSA in PBST FL: 1:500 in 5% BSA CF: 1:200 in blocking solution	Cell Signaling Technology, Danvers, MA, USA
TRX	14999-1-AP	Polyclonal/rabbit	WB: 1:10,000 in 5% BM in PBST FL: 1:1,000 in 5% BSA CF: 1:400 in blocking solution	Proteintech, Planegg-Martinsried, Germany

WB = Western blotting, FL = fluorometry, CF = confocal microscopy, PBST = phosphate-buffered saline + tween-20, BM =  blocking milk, BSA = bovine serum albumine.

### Fluorometry

Fluorometry was used to investigate the effects of the different inhibitors on extracellular TG2-mediated cross-linking of the TG2 substrate 5-(biotinamido)-pentylamine (5BP; 500 µM; Thermo Fisher Scientific) after the Caco-2 cells had been stimulated with 1,000 IU/mL of IFN-γ (Sigma-Aldrich) for 24 h.

In addition, the protein expression of ERp57, TRX, and TG2 as well as the TG2 activity in the Caco-2 cells was investigated fluorometrically after siRNA transfection.

After the end of the IFN-γ or siRNA treatment, the Caco-2 cells were incubated with 5BP at 37°C for 3 h. To investigate the intracellular protein expression and TG2 activity, cells were permeabilized with 0.5% Triton for 5 min after fixation with 4% paraformaldehyde. This step was omitted when the extracellular protein expression and TG2 activity were investigated. Cells were blocked with 5% bovine serum albumin and incubated with the corresponding primary antibodies directed against ERp57, TRX, or TG2 at 4°C overnight on an orbital shaker (only siRNA experiments, Table 3). After three washing steps in phosphate buffered saline (PBS), the secondary antibody (goat anti-rabbit Alexa555, Thermo Fisher Scientific) was added. To visualize the TG2-mediated cross-linking of 5BP, streptavidin-Alexa488 (2 µg/mL, Thermo Fisher Scientific) was applied at room temperature for 1 h. Fluorometric quantitation was performed by using a Clariostar Plus microplate reader.

### Confocal microscopy

Human duodenal biopsies were fixed in 5% paraformaldehyde in PIPES buffer, placed in polyvinylpyrrolidone sucrose and frozen in liquid nitrogen. Cryosections (400 nm) were obtained by using a cryo-ultramicrotome (Leica EM UC6, Leica, Wetzlar, Germany). The permeabilization and blocking of tissue sections has been described elsewhere [12]. Incubation with the primary antibody diluted in blocking solution was performed overnight at 4°C (Table 3). AlexaFluor-488-conjugated secondary antibody (goat anti-rabbit; 1:200; Thermo Fisher Scientific) was applied for 1 h in the dark the next day. Nuclei were stained by using Hoechst dye (Thermo Fisher Scientific) diluted in PBS (1:1,000) for 10 min. Imaging was performed by using a confocal laser scanning microscope (TE2000-E, Nikon, Langen, Germany) and a 60x Plan Apo (NA 1.41) immersion oil objective. Six images per condition were obtained (FITC channel: 488/496 nm, UV-channel: 352/454 nm). Image analysis was performed by using Image J [35]. The mean fluorescence intensity of the FITC channel was quantified after the regions of interest in the epithelium and the *lamina propria* were separately drawn manually.

### Statistics

Statistical analysis was performed by using GraphPad Prism (version 10.2.2 GraphPad Prism Software Inc., San Diego, CA, USA). Student’s unpaired two-tailed *t*-test (with Welch correction where appropriate) or *U* test were used where appropriate. *P* values of <0.05 were considered significant.

## Results

### Active-site and allosteric inhibitors reduce cross-linking of P56-88 by recombinant TG2

**Figure 1.**
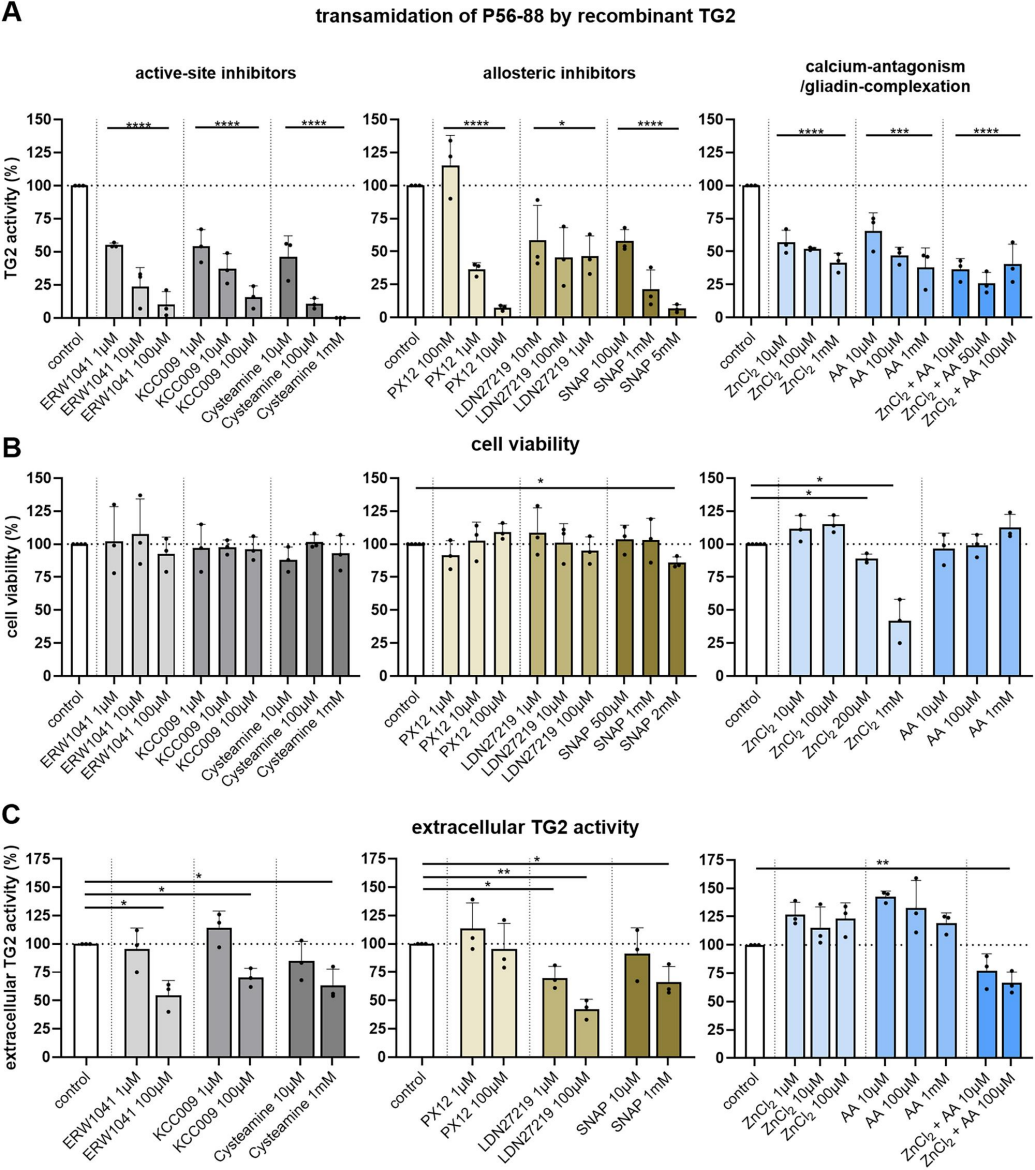
Effects of different TG2 inhibitors on the cross-linking activity of recombinant TG2, cell viability, and extracellular TG2 activity. (A) Fluorometric quantitation of the cross-linking of biotin-conjugated gliadin peptide P56-88 by recombinant TG2 (*n = *3, three technical replicates per experiment). (B) Fluorometric quantitation of cell viability after 24 h of incubation with the corresponding substances using the resazurin-based assay (*n = *3, two technical replicates per experiment). (C) Fluorometric quantitation of extracellular cross-linking of the TG2 substrate 5BP by Caco-2 cells (*n = *3, with three technical replicates per experiment). All data are shown as mean ± SD. Statistical significance was tested by using univariate ANOVA (A) or Student’s *t*-test with Welch correction. *P < 0.05, **P < 0.01, ***P < 0.001, ****P < 0.0001. 5BP = 5-(biotinamido)-pentylamine, ZnCl_2_ = zinc chloride, AA = ascorbic acid.

First, we investigated the effects of different inhibitory approaches on the cross-linking of the gliadin peptide P56-88 by recombinant TG2. All tested inhibitors were effective and reduced TG2-mediated transamidation in a concentration-dependent manner. The active-site-directed inhibitors ERW1041, KCC009, and cysteamine had comparable effects and reduced TG2 activity by ∼90% at 100 µM (ERW1041 (10 ± 10)%, KCC009 (16 ± 9)%, cysteamine (11 ± 4)%, Figure 1A). As allosteric inhibitors, PX12 markedly reduced the TG2 activity at low concentrations of ∼10 µM (7 ± 2)%, whereas LDN27219 only decreased the cross-linking by ∼50% at the doses tested. SNAP also significantly reduced the TG2 activity, but only at high concentrations of >1 mM (Figure 1A). Zinc chloride and ascorbic acid had moderate effects and reduced the TG2 activity by ∼60% at the highest concentrations (1 mM, Figure 1A). The equimolar combination of zinc chloride and ascorbic acid (10 µM) was significantly more efficient than the treatment with each substance alone (*P < *0.05, Figure 1A). At the higher concentration of 100 µM, there was no difference between the combination of zinc chloride and ascorbic acid and the application of both substances alone.

### Active-site and allosteric inhibitors reduce extracellular TG2 activity in Caco-2 cells

Based on the results of the experiments with recombinant TG2 and the existing literature, we selected the inhibitor concentrations for the cell culture experiments. We performed a resazurin-based assay to investigate their impact on the viability of Caco-2 cells. Most substances did not have any effects on cell survival in the doses used in the experiments described above (Figure 1B). Only SNAP [(86 ± 4)% at 2 mM, (54 ± 12)% at 5 mM, *P *< 0.05, Figure 1B] and zinc chloride [(89 ± 4)% at 200 µM, (42 ± 17)% at 1 mM, *P *< 0.05, Figure 1B] negatively affected cell viability at higher concentrations. Therefore, inhibitor doses were adjusted to investigate the inhibitory potential of the drug candidates on extracellular TG2 on Caco-2 cell monolayers.

The active-site inhibitors ERW1041, KCC009, and cysteamine reduced extracellular TG2 activity by ∼30%–40% (Figure 1C). In the group of allosteric inhibitors, PX12 did not affect extracellular TG2 activity, whereas LDN27219 reduced the cross-linking of 5BP in a dose-dependent manner [(70 ± 10)% at 10 µM, *P *< 0.05, (42 ± 9)% at 100 µM, *P *< 0.01, Figure 1C). SNAP also inhibited the TG2 activity but only at high concentrations of ∼1 mM (66 ± 32)% (*P *< 0.05). Zinc chloride and ascorbic acid alone did not affect the extracellular cross-linking of 5BP, but, when they were combined, we observed a dose-dependent inhibitory effect [(66 ± 10)% at 100 µM, *P *< 0.01, Figure 1C].

### IFN-γ stimulation does not affect epithelial expression of ERp57 and TRX

**Figure 2.**
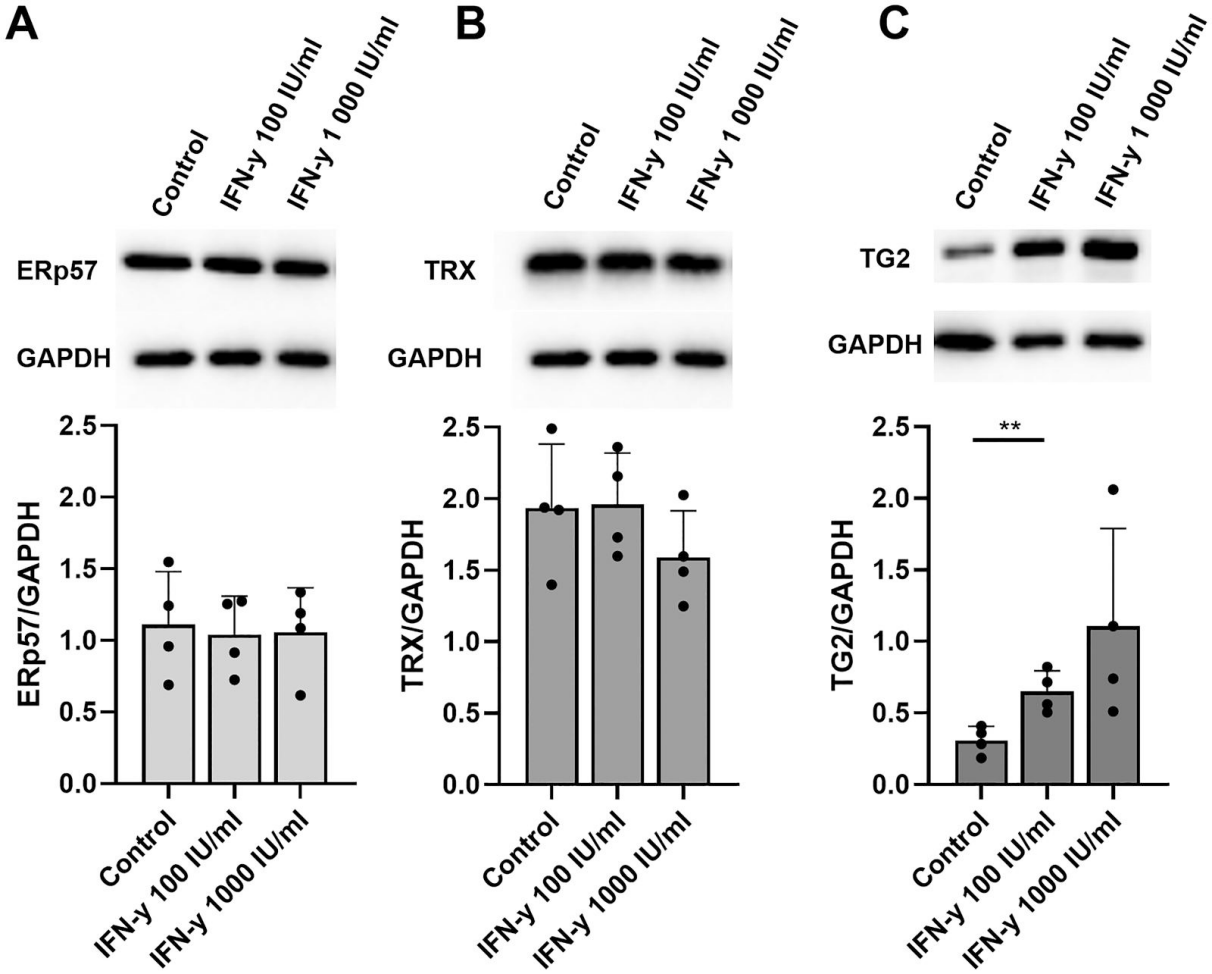
Effect of IFN-γ on the expression of ERp57, TRX and TG2 in Caco-2 cells. Analysis of relative expression of (A) ERp57, (B) TRX, and (C) TG2 by Western blotting after stimulation with different concentrations of IFN-γ over 48 h. No significant change in the expression of ERp57 and TRX. Dose-dependent increase in TG2 expression. All data are shown as mean ± SD. Statistical significance was tested by using Student’s *t*-test. *n = *4. *P < 0.05.

We further aimed to clarify the role of the two endogenous oxidative proteins ERp57 and TRX. First, we investigated whether the expression of both proteins was associated with a Th1-dominated immune response of CD. For this purpose, we treated Caco-2 cells with IFN-γ, the main cytokine of Th1-inflammation. Stimulation with different concentrations of IFN-γ over 48 h did not significantly affect the expression of ERp57 or TRX (Figure 2A and B). In contrast, the TG2 expression significantly increased by ∼200% and ∼400% when using 100 and 1,000 IU/mL of IFN-γ, respectively (Figure 2C).

### SiRNA-mediated knockdown of *TRX* reduces cellular TG2 activity

**Figure 3.**
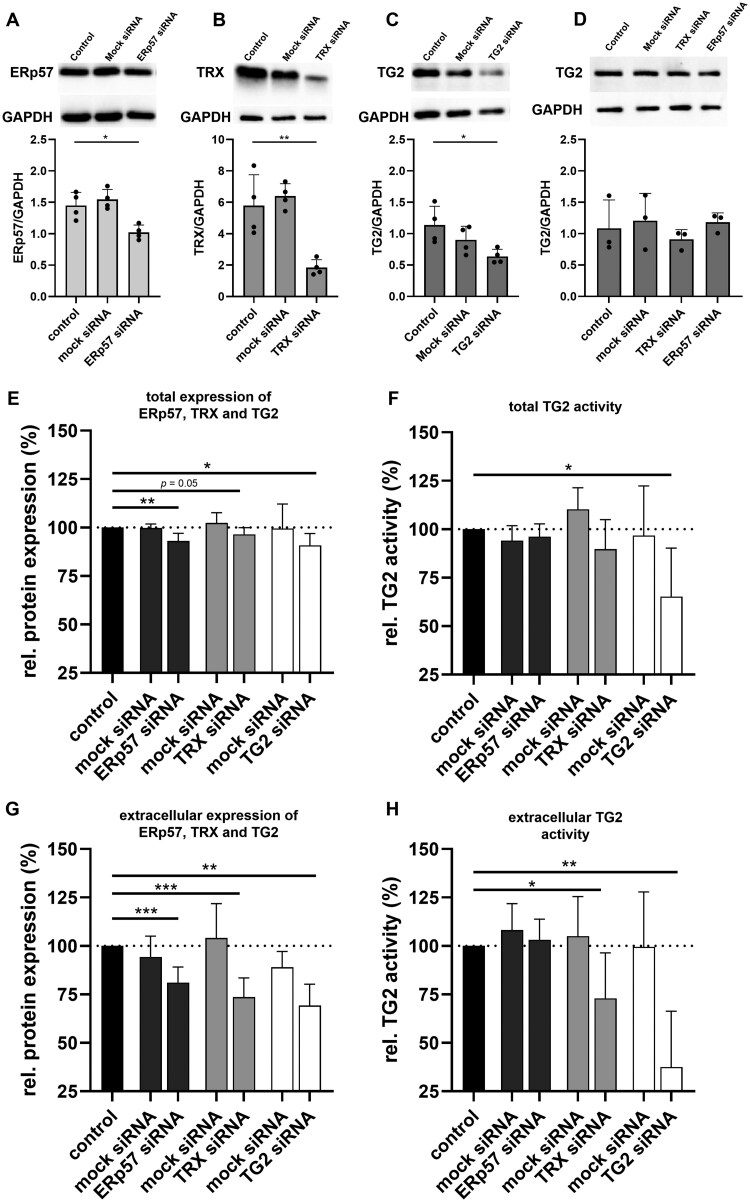
SiRNA knockdown of ERp57, TRX, and TG2, and the effect on cellular/extracellular TG2 activity. Caco-2 cells were incubated with specific siRNAs or mock siRNA for 48 h. The efficacy of the siRNA knockdown was determined by using Western blotting. Expression of ERp57, TRX, and TG2 was normalized against the housekeeping protein GAPDH. (A–C) SiRNA treatment significantly reduced expression of (A) ERp57, (B) TRX, and (C) TG2. Incubation with mock siRNA did not affect protein expression (*n = *4). (D) Treatment with ERp57-siRNA und TRX-siRNA did not show any off-target effects on TG2 expression (*n = *3). (E) Cellular expression of ERp57, TRX, and TG2 determined by fluorometry after siRNA knockdown. Protein levels of all three target proteins were reduced by siRNA-mediated knockdown. (F) Cellular TG2 activity was determined by fluorometry after siRNA treatment. SiRNA knockdown of TRX and TG2 reduced cellular TG2 activity (*n = *5). (G) Extracellular expression of ERp57, TRX, and TG2 was investigated in unpermeabilized Caco-2 cells by fluorometry. Protein levels of all three target proteins were significantly reduced by siRNA-mediated knockdown. (H) Extracellular TG2 activity was investigated in unpermeabilized Caco-2 cells by fluorometry. SiRNA knockdown of TRX and TG2 significantly reduced extracellular TG2 activity (*n = *5). All data are shown as mean ± SD. Statistical significance was tested by using Student’s *t*-test with Welch correction where appropriate. *P < 0.05, **P < 0.01, ***P < 0.001. GAPDH = glyceraldehyde 3-phosphate dehydrogenase.

To investigate the effect of ERp57 and TRX on cellular TG2 activity, we used siRNA knockdown in Caco-2 cells and observed a reduced expression of ERp57 by ∼30% (1.5 ± 0.2 vs 1.0 ± 0.1; *P *< 0.05, Figure 3A) compared with the control condition. The most effective knockdown by 70% (5.8 ± 2.0 vs 1.8 ± 0.5; *P *< 0.01, Figure 3B) was shown for TRX, but the TG2 expression was also reduced by 45% (1.1 ± 0.3 vs 0.6 ± 0.1; *P *< 0.05, Figure 3C). The treatment of Caco-2 cells with mock siRNA did not significantly affect the expression of ERp57, TRX, or TG2 (Figure 3A–C). To exclude unspecific effects of the siRNAs targeting ERp57 and TRX on the TG2 expression, we further analysed the protein levels of TG2 after siRNA treatment. Incubation with siRNAs targeting ERp57 and TRX did not affect the protein levels of TG2 (Figure 3D). Next, we investigated the expression of ERp57, TRX, and TG2 as well as TG2 activity in permeabilized Caco-2 cells by using fluorometry and detected consistent effects of the siRNA treatment on the total cellular expression of all three target proteins. ERp57 expression was reduced by ∼7% [(93 ± 4)%; *P *< 0.01, Figure 3E] compared with the control condition. We also observed a trend towards a reduced expression of TRX [(96 ± 4)%, *P *= 0.05, Figure 3E], but the most prominent reduction was observed for the TG2 expression [(91 ± 6)%, *P *< 0.05, Figure 3E].

TG2 activity was measured concomitantly by cross-linking of the substrate 5BP. Incubation with *ERp57*- and *TRX*-siRNA did not significantly alter the TG2 activity (Figure 3F). SiRNA-mediated knockdown of TG2, however, significantly reduced the TG2 activity by 35% [(65 ± 25)%, *P *< 0.05, Figure 3F] compared with controls. Treatment with mock siRNA did not affect the protein expression of ERp57, TRX, and TG2, nor did it influence the TG2 activity (Figure 3E and F).

### SiRNA-mediated knockdown of TRX reduces extracellular TG2 activity

Extracellular TG2 activity, considered to be a key factor in CD pathogenesis, was determined by using the fluorometric assay described above, but without cell permeabilization.

In line with the previous experiments, the extracellular levels of all target proteins were significantly reduced after siRNA knockdown, i.e. the expression of ERp57 was reduced by 19% [(81 ± 8)%, *P *< 0.01, Figure 3G] and the TRX expression by 26% [(74 ± 10)%, *P *< 0.01, Figure 3G]. The most prominent effect was observed for TG2, which was reduced by 31% [(69 ± 4)%, *P *< 0.01, Figure 3G]. Treatment with ERp57-siRNA did not affect the extracellular TG2 activity, whereas the treatment with TRX-siRNA led to a reduction by 27% [(73 ± 23)%, *P *< 0.05, Figure 3H]. Incubation with TG2-siRNA resulted in a reduction of extracellular TG2 activity by ∼63% [(37 ± 29)%, *P *< 0.01, Figure 3G]. Again, treatment with mock siRNA did not influence the extracellular expression of ERp57, TRX, and TG2 or the TG2 activity (Figure 3G and H).

### The expression of ERp57, TRX, and TG2 is increased in the duodenal mucosa of CD patients

To further address the functional relevance of ERp57 and TRX in CD, we evaluated the expression of both proteins in duodenal tissue samples obtained from control and CD patients.

**Figure 4.**
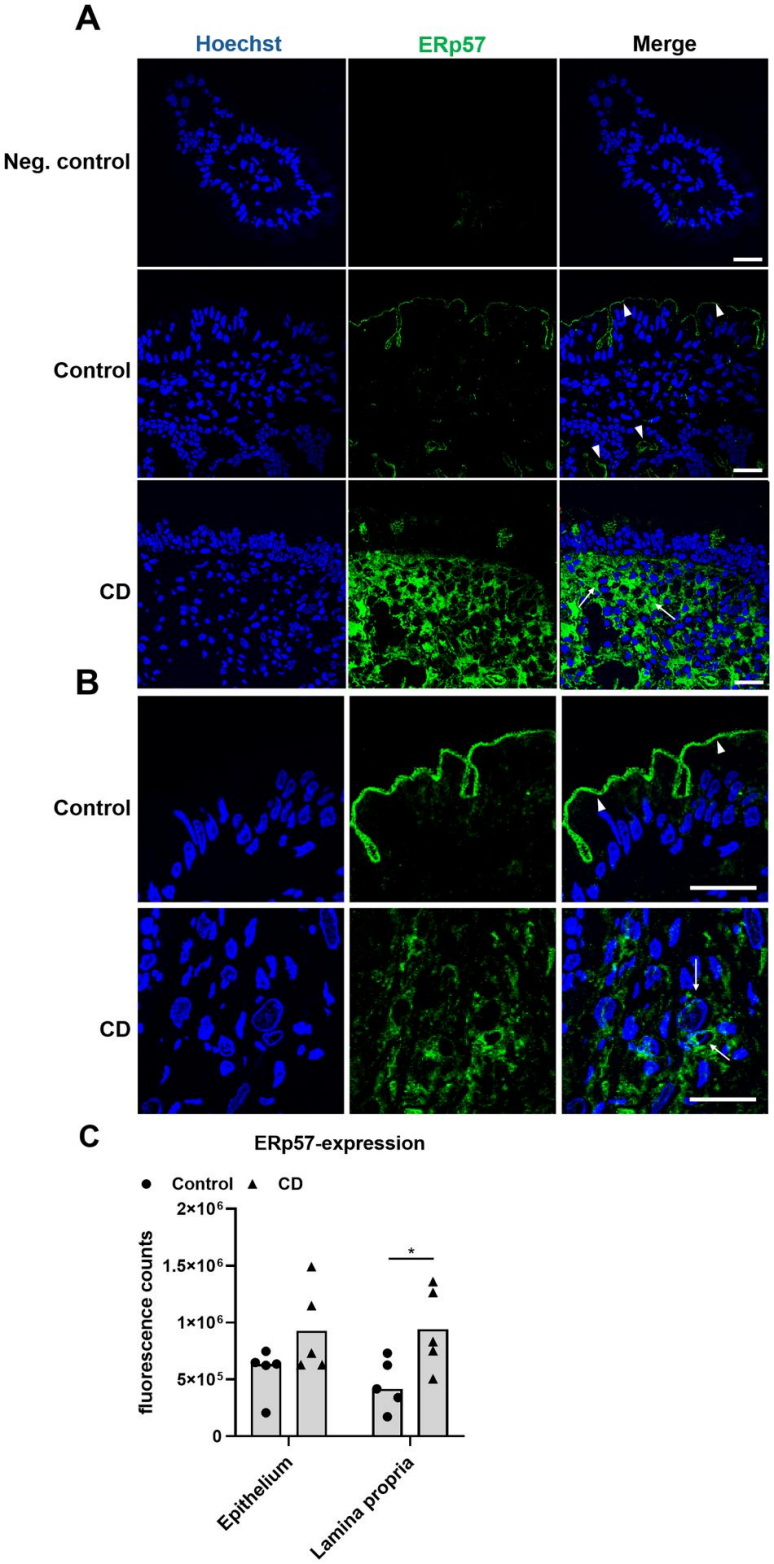
Intestinal expression of ERp57 in control and CD patients. (A) Confocal microscopy of ERp57 in duodenal cryosections of control and CD patients revealed its predominant expression at the BBM (arrowheads) of control patients and the intestinal *lamina propria* (arrows) of CD patients. (B) Detailed images of the duodenal mucosa reveal predominant expression of ERp57 at the BBM (arrowheads) of control patients. In CD biopsies, ERp57 was strongly expressed in the *lamina propria* (arrows). (C) Overall, expression of ERp57 was significantly higher in the *lamina propria* of CD patients compared with those in controls. All data are shown as median. Statistical significance was tested by using Student’s *t*-test. *n =* 5. *P < 0.05; scale: 20 µm. BBM = brush border membrane.

The expressions of ERp57 in the duodenal mucosa differed between the control and CD patients. In the control patients, ERp57 was primarily observed at the brush border membrane (Figure 4A–C), whereas the mucosa from CD patients revealed a significantly stronger expression of ERp57 in the *lamina propria* (Figure 4A–C).

**Figure 5.**
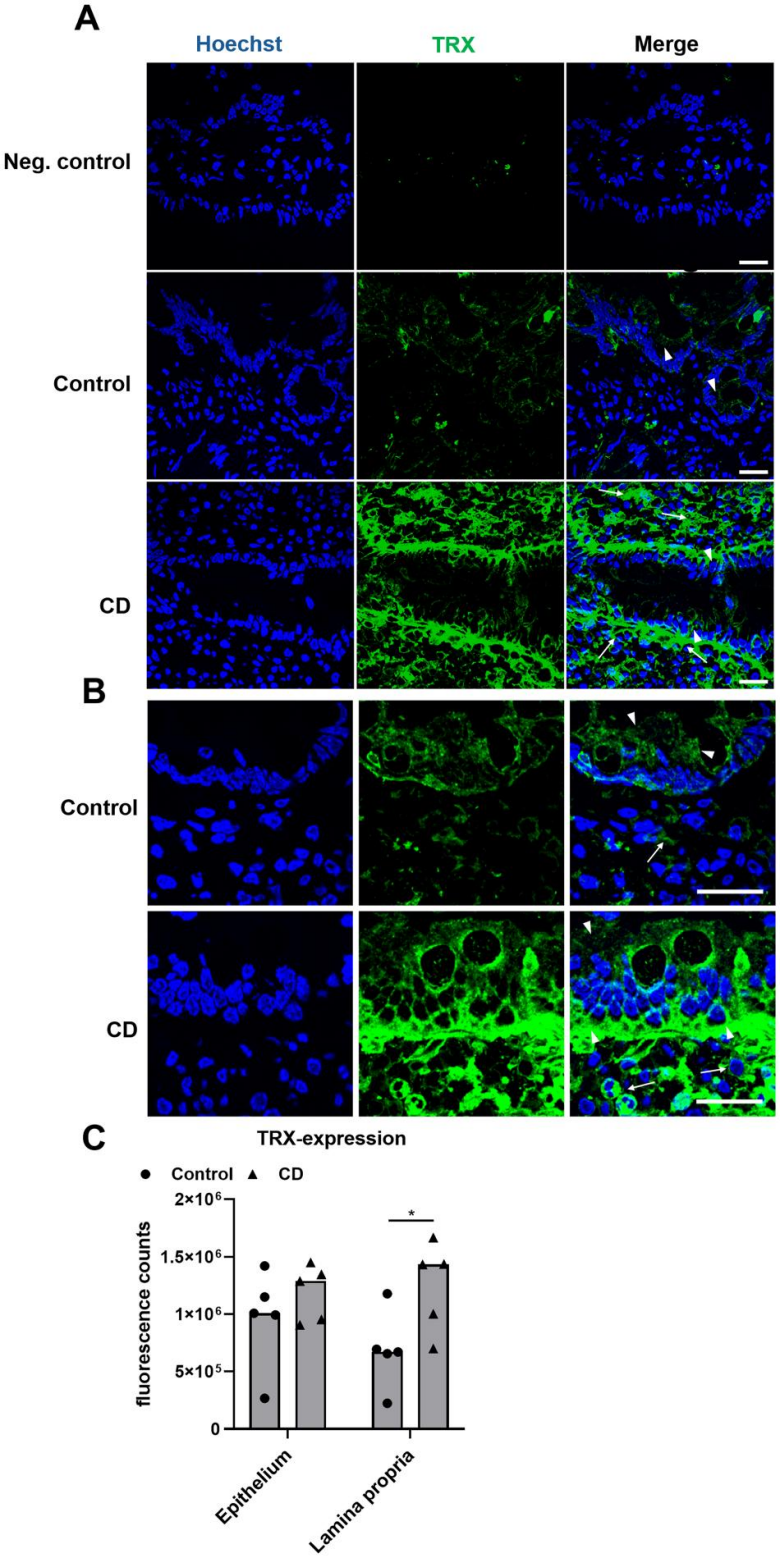
Expression of TRX is significantly increased in CD biopsies. (A) In control patients, TRX was slightly expressed in the epithelium (arrowheads), but not in the *lamina propria*. In CD biopsies, prominent levels of TRX were observed in the *lamina propria* (arrows) and the basal epithelium (arrowheads). (B) Detailed images of the duodenal mucosa reveal predominant expression of TRX in the epithelium (arrowheads) of control patients and low levels in the *lamina propria* (arrows). In CD biopsies, TRX was strongly expressed in the basal epithelium (arrowheads) and the *lamina propria* (arrows). (C) Expression of TRX was significantly higher in the *lamina propria* of CD biopsies compared with those of controls. All data are shown as median. Statistical significance was tested by using Student’s *t*-test. *n = *5. *P < 0.05; scale: 20 µm.

In addition, we investigated the expression of TRX in control and CD duodenal biopsies. In the control patients, we observed low levels of TRX in the basal epithelium and the *lamina propria* (Figure 5A–C), whereas, in the CD patients, TRX expression in the *lamina propria* was significantly higher compared with that in the controls (Figure 5A–C).

**Figure 6.**
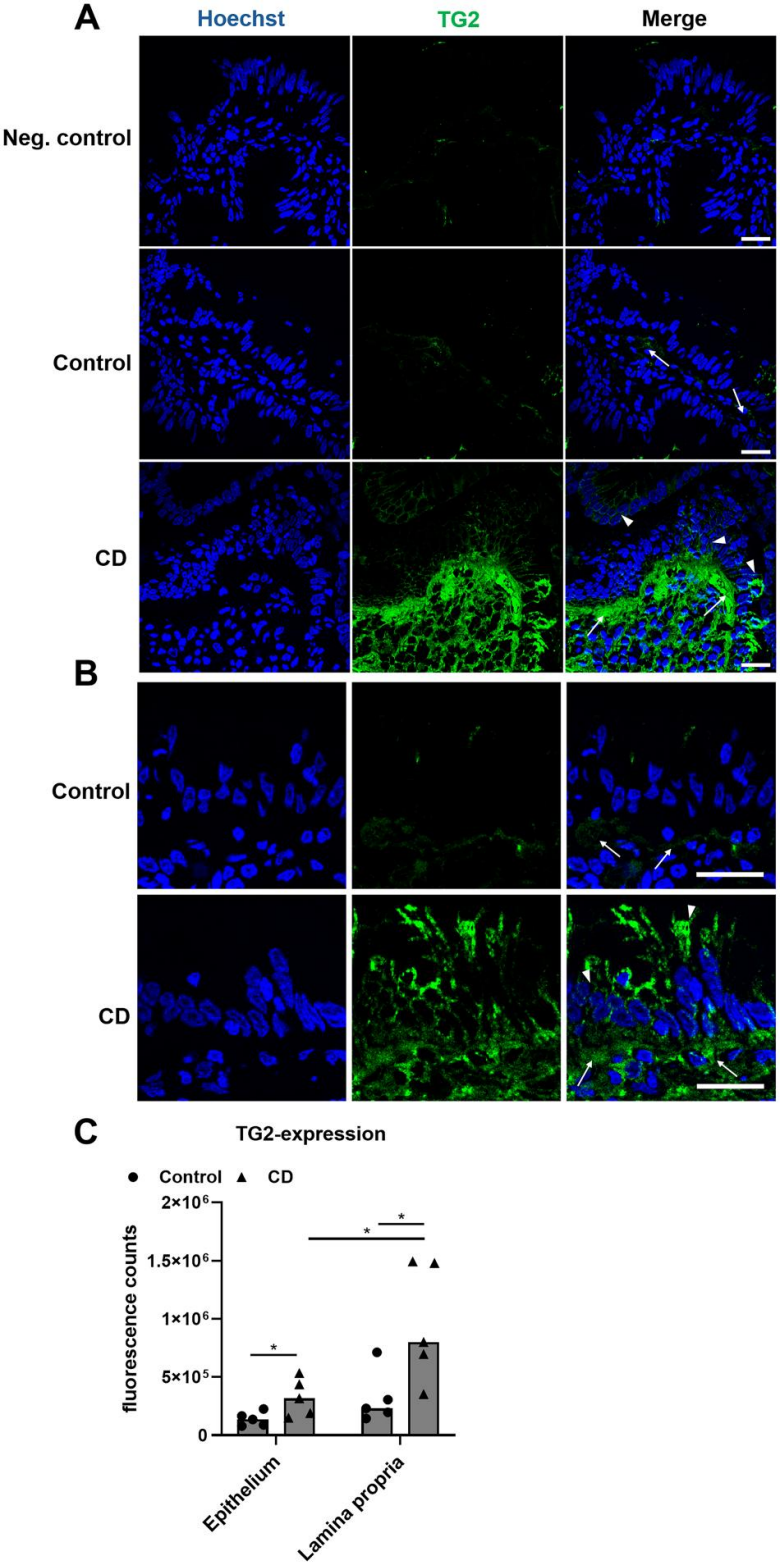
TG2 is expressed in the *lamina propria* and the epithelium of CD biopsies. (A) Fluorescence staining revealed TG2 to be nearly absent in the epithelium of control biopsies and low levels were expressed in the *lamina propria* (arrows). In CD biopsies, TG2 expression was increased in the epithelium (arrowheads) and the *lamina propria* (arrows). (B) Detailed images of the duodenal mucosa reveal high levels of TG2 in the *lamina propria* (arrows) and the epithelium (arrowheads) of CD patients. (C) TG2 expression was significantly increased in the epithelium and the *lamina propria* of CD patients. All data are shown as median. Statistical significance was tested by using Student’s *t*-test. *n = *5. *P < 0.05; scale: 20 µm.

Regarding TG2, significant differences were observed for its expression sites when duodenal biopsies were evaluated by using fluorescence staining. As expected, the extracellular TG2 expression was increased in the CD biopsies (Figure 6A–C). Here, TG2 was present on the cell surface of duodenal enterocytes (Figure 6A and B). In four out of five control patients, however, we did not observe any significant TG2 expression in the epithelium. Staining of the TG2 was present in the *lamina propria* of the control patients, but to a much lower extent compared with that in the CD patients (Figure 6A–C).

## Discussion

In this study, we aimed to compare the effects of different approaches of TG2 inhibition and their applicability in a cell culture model.

First, we investigated the effects of different inhibitory mechanisms on the activity of recombinant and extracellular TG2. Besides the TG2-specific, active-site-directed inhibitors ERW1041 and KCC009, we also tested the competitive inhibitor cysteamine as well as the allosteric inhibitors PX12, LDN27219, and SNAP, in addition to zinc chloride (calcium antagonist) and ascorbic acid (gliadin complexation). All substances significantly reduced cross-linking of the gliadin peptide P56-88 by recombinant TG2 in a dose-dependent manner. The active-site-directed inhibitors showed comparable results at doses of 100 µM (reduction by ∼90%). Among the allosteric inhibitors, PX12 and SNAP markedly reduced TG2-mediated cross-linking, but, for SNAP, high concentrations of 1 mM were needed. Zinc chloride and ascorbic acid alone only had moderate effects on TG2-mediated cross-linking. The combination of both substances, however, increased the TG2 inhibition, but, still, the influence was less efficient compared with those of the active-site inhibitors, with a reduction of 30%–50%. In the cell culture model, LDN27219 (reduction by 50%) and SNAP also showed promising results, but, for SNAP, again high doses close to cytotoxic concentrations were needed. PX12 did not affect TG2 activity in the cell culture experiments, as described before [12]. Finally, we did not observe any effect of zinc chloride and ascorbic acid alone on the extracellular TG2 activity. The combination of both only resulted in a moderate inhibition at zinc chloride doses close to cytotoxic concentrations.

Recent observations indicated that the addition of zinc chloride and ascorbyl palmitate to wheat flour might prevent the TG2-mediated modification of gliadin peptides [22, 23]. However, the low efficiency of both substances in our cell culture experiments argues against this option. The application of LDN27219 and SNAP have been investigated by using different cell culture and *ex vivo* models [19, 21, 36], but this is the first study to have investigated their potential in the context of CD. Altogether, the TG2-specific active-site inhibitors ERW1041 and KCC009 showed the most consistent and prominent results. This observation is in line with the findings of a Phase II clinical trial in which the TG2-specific inhibitor ZED1227 attenuated gluten-induced mucosal damage in CD patients [16]. However, in contrast to those active-site-directed inhibitors, application of the allosteric inhibitor LDN27219 might have several advantages. LDN27219 induces a conformational change in TG2, which might also target the non-catalytic functions of TG2 that may also play a role in CD [19]. Furthermore, LDN27219 might have fewer off-target effects, as allosteric sites tend to be more unique than active sites. Finally, the allosteric inhibition is potentially reversible, allowing finely tuned regulation of the TG2 activity [37].

In addition, our data indicate that the siRNA-mediated knockdown of TG2 might represent an alternative approach, leading to the most effective reduction of extracellular TG2 activity. This is in line with data from a murine model that demonstrated the effect of intravenous siRNA application on TG2 expression in pancreatic tumor cells [38]. Even though several siRNA-based therapies have already reached clinical practice, this approach still faces several limitations in the context of CD, such as the tropism of the molecule, its stability, and off-target effects [39, 40].

Finally, we also focused on the therapeutic potential of the regulatory proteins ERp57 and TRX by directly addressing their impact on cellular and extracellular TG2 activity. In our study, the siRNA-mediated knockdown of ERp57 did not affect cellular or extracellular TG2 activity (Figure 3), which is in contrast to the data from *Yi et al.*, who described a possible inhibitory role of ERp57 on extracellular TG2 activity in human umbilical vein endothelial cells [32]. For the TRX-siRNA treatment, however, we observed a reduced cellular and extracellular TG2 activity, confirming the activating effect of TRX. This was in agreement with existing literature that has shown the stimulating effect of exogenous TRX on extracellular TG2 activity on colonic epithelial monolayers, macrophages, fibroblasts, and duodenal tissue sections from mice [24–26]. The differential effect observed between ERp57 and TRX might be attributable to the larger redox potential difference between TRX (–230 mV) and TG2 (–190 mV) compared with that between ERp57 (–167mV) and TG2 [32, 41, 42]. However, the lower knockdown-efficiency of ERp57-siRNA compared with TRX-siRNA might be an alternative explanation.

The potential role of TRX in the intestinal mucosa was also suggested by our finding of an increased TRX expression in the *lamina propria* of CD biopsies (Figure 5). As we did not observe an effect of IFN-γ stimulation on the TRX expression in Caco-2 cells, the TRX in the mucosa probably derived from fibroblasts or macrophages and not from enterocytes [24, 25, 43]. However, we also observed an increased expression of ERp57 in the *lamina propria* of CD patients (Figure 4). Thus, one might assume that CD-associated inflammation may actually trigger the release of TRX and ERp57 by fibroblasts or macrophages [24, 43].

In this context, we also observed significant epithelial levels of TG2 in CD biopsies. Even though TG2 is suggested to exert its pathogenetic role in the *lamina propria*, several studies also pointed towards a contribution of epithelial TG2 to the transport of gliadin peptides [12, 44]. This was supported by our observation of the TG2 expression on the cell membranes of duodenal enterocytes in the CD biopsies.

## Conclusions

Altogether, our data underline the hypothesis that increased TRX expression might contribute to CD pathogenesis by activating TG2. Furthermore, we demonstrated the high efficiency of TG2-specific active-site inhibitors and the siRNA-mediated knockdown of TG2 on extracellular TG2 activity. Future research is needed to investigate the potential side effects of long-term inhibition of TG2 activity and the applicability of siRNA-based therapy for CD.

## Authors’ contributions

S.S. conceived and designed the project and performed data curation, interpreted the data, and wrote the manuscript. S.R., J.D.L., and K.-P.Z. supported the conceptualization and data interpretation, and reviewed the manuscript. A.E. and P.G. performed the experiments, analysed the data, and supported the writing of the manuscript. All authors read and approved the final manuscript.

## References

[1] Iversen R , Sollid LM. The immunobiology and pathogenesis of celiac disease. Annu Rev Pathol Mech Dis 2023;18:47–70. 10.1146/annurev-pathmechdis-031521-032634 36067801

[2] Arentz-Hansen H , Korner R, Molberg O et al The intestinal T cell response to alpha-gliadin in adult celiac disease is focused on a single deamidated glutamine targeted by tissue transglutaminase. J Exp Med 2000;191:603–12. 10684852 10.1084/jem.191.4.603 PMC2195837

[3] Shan L , Molberg O, Parrot I et al Structural basis for gluten intolerance in Celiac sprue. Science (1979) 2002;297:2275–9. 10.1126/science.1074129 12351792

[4] Molberg Ø , McAdam SN, Korner R et al Tissue transglutaminase selectively modifies gliadin peptides that are recognized by gut-derived T cells in celiac disease. Nat Med 1998;4:713–7. 9623982 10.1038/nm0698-713

[5] Levescot A , Malamut G, Cerf-Bensussan N. Immunopathogenesis and environmental triggers in coeliac disease. Gut 2022;71:2337–49. 35879049 10.1136/gutjnl-2021-326257 PMC9554150

[6] Schuppan D , Ciccocioppo R. Coeliac disease and secondary autoimmunity. Dig Liver Dis 2002;34:13–5. 11926568 10.1016/s1590-8658(02)80053-6

[7] Rauhavirta T , Hietikko M, Salmi T et al Transglutaminase 2 and transglutaminase 2 autoantibodies in celiac disease: a review. Clin Rev Allergy Immunol 2019;57:23–38. 27263022 10.1007/s12016-016-8557-4

[8] Tatsukawa H , Hitomi K. Role of transglutaminase 2 in cell death, survival, and fibrosis. Cells 2021;10:1842. 34360011 10.3390/cells10071842 PMC8307792

[9] Klöck C , Khosla C. Regulation of the activities of the mammalian transglutaminase family of enzymes. Protein Sci 2012;21:1781–91. 23011841 10.1002/pro.2162 PMC3575910

[10] Stamnaes J , Pinkas DM, Fleckenstein B et al Redox regulation of transglutaminase 2 activity. J Biol Chem 2010;285:25402–9. 20547769 10.1074/jbc.M109.097162 PMC2919103

[11] Abadie V , Kim SM, Lejeune T et al IL-15, gluten and HLA-DQ8 drive tissue destruction in coeliac disease. Nature 2020;578:600–4. 32051586 10.1038/s41586-020-2003-8 PMC7047598

[12] Stricker S , de Laffolie J, Zimmer K-P et al Inhibition of transglutaminase 2 as a therapeutic strategy in celiac disease—in vitro studies in intestinal cells and duodenal biopsies. IJMS 2023;24:4795. 36902226 10.3390/ijms24054795 PMC10002517

[13] Yuan L , Choi K, Khosla C et al Transglutaminase 2 inhibitor, KCC009, disrupts fibronectin assembly in the extracellular matrix and sensitizes orthotopic glioblastomas to chemotherapy. Oncogene 2007;26:2563–73. 17099729 10.1038/sj.onc.1210048

[14] Jeon J-H , Lee H-J, Jang G-Y et al Different inhibition characteristics of intracellular transglutaminase activity by cystamine and cysteamine. Exp Mol Med 2004;36:576–81. 15675041 10.1038/emm.2004.74

[15] Dafik L , Albertelli M, Stamnaes J et al Activation and Inhibition of Transglutaminase 2 in Mice. PLoS One 2012;7:e30642. 22319575 10.1371/journal.pone.0030642 PMC3271093

[16] Schuppan D , Mäki M, Lundin KEA et al; CEC-3 Trial Group. A randomized trial of a transglutaminase 2 inhibitor for celiac disease. N Engl J Med 2021;385:35–45. 34192430 10.1056/NEJMoa2032441

[17] Büchold C , Hils M, Gerlach U et al Features of ZED1227: the first-in-class tissue transglutaminase inhibitor undergoing clinical evaluation for the treatment of celiac disease. Cells 2022;11:1677. 35626704 10.3390/cells11101667 PMC9139979

[18] Stricker S , Rudloff S, De Laffolie J et al Tissue transglutaminase but not microbial transglutaminase is inhibited by exogenous oxidative substances in celiac disease. Int J Mol Sci 2022;23:2248. 35216364 10.3390/ijms23042248 PMC8879474

[19] Pinilla E , Comerma-Steffensen S, Prat-Duran J et al Transglutaminase 2 inhibitor LDN 27219 age-dependently lowers blood pressure and improves endothelium-dependent vasodilation in resistance arteries. Hypertension 2021;77:216–27. 33249864 10.1161/HYPERTENSIONAHA.120.15352

[20] Telci D , Collighan RJ, Basaga H et al Increased TG2 expression can result in induction of transforming growth factor beta1, causing increased synthesis and deposition of matrix proteins, which can be regulated by nitric oxide. J Biol Chem 2009;284:29547–58. 19657147 10.1074/jbc.M109.041806 PMC2785588

[21] van den Akker J , VanBavel E, van Geel R et al The redox state of transglutaminase 2 controls arterial remodeling. PLoS ONE 2011:6:e23067. 21901120 10.1371/journal.pone.0023067 PMC3161997

[22] Engstrom N , Saenz-Méndez P, Scheers J et al Towards Celiac-safe foods: decreasing the affinity of transglutaminase 2 for gliadin by addition of ascorbyl palmitate and ZnCl2 as detoxifiers. Sci Rep 2017;7:77. 28250436 10.1038/s41598-017-00174-z PMC5427931

[23] Engström N , Böhn L, Josefsson A et al Development of celiac-safe foods: prevention of transglutaminase 2 (TG2) deamidation of gluten in healthy non-celiac volunteers. Front Nutr 2024;11:1308463. 38549745 10.3389/fnut.2024.1308463 PMC10972847

[24] Jin X , Stamnaes J, Klöck C et al Activation of extracellular transglutaminase 2 by thioredoxin. J Biol Chem 2011;286:37866–73. 21908620 10.1074/jbc.M111.287490 PMC3199528

[25] Plugis NM , Palanski BA, Weng C-H et al Thioredoxin-1 selectively activates transglutaminase 2 in the extracellular matrix of the small intestine: implications for celiac disease. J Biol Chem 2017;292:2000–8. 28003361 10.1074/jbc.M116.767988 PMC5290969

[26] DiRaimondo TR , Plugis NM, Jin X et al Selective inhibition of extracellular thioredoxin by asymmetric disulfides. J Med Chem 2013;56:1301–10. 23327656 10.1021/jm301775s PMC3574193

[27] Liu Y , Xue N, Zhang B et al Role of Thioredoxin-1 and its inducers in human health and diseases. Eur J Pharmacol 2022;919:174756. 35032486 10.1016/j.ejphar.2022.174756

[28] Shcholok T , Eftekharpour E. Insights into the multifaceted roles of thioredoxin-1 system: exploring knockout murine models. Biology (Basel) 2024;13:180. 38534450 10.3390/biology13030180 PMC10968256

[29] Oberacker T , Kraft L, Schanz M et al The importance of thioredoxin-1 in health and disease. Antioxidants (Basel) 2023;12:1078. 37237944 10.3390/antiox12051078 PMC10215468

[30] Bertini R , Howard OMZ, Dong H-F et al Thioredoxin, a redox enzyme released in infection and inflammation, is a unique chemoattractant for neutrophils, monocytes, and T cells. J Exp Med 1999;189:1783–9. 10359582 10.1084/jem.189.11.1783 PMC2193090

[31] Mougiakakos D , Johansson CC, Jitschin R et al Increased thioredoxin-1 production in human naturally occurring regulatory T cells confers enhanced tolerance to oxidative stress. Blood 2011;117:857–61. 21030559 10.1182/blood-2010-09-307041

[32] Yi MC , Melkonian AV, Ousey JA et al Endoplasmic reticulum-resident protein 57 (ERp57) oxidatively inactivates human transglutaminase 2. J Biol Chem 2018;293:2640–9. 29305423 10.1074/jbc.RA117.001382 PMC5827427

[33] Chichiarelli S , Altieri F, Paglia G et al ERp57/PDIA3: new insight. Cell Mol Biol Lett 2022;27:12. 35109791 10.1186/s11658-022-00315-x PMC8809632

[34] Husby S , Koletzko S, Korponay-Szabó I et al European society paediatric gastroenterology, hepatology and nutrition guidelines for diagnosing coeliac disease 2020. J Pediatr Gastroenterol Nutr 2020;70:141–56. 31568151 10.1097/MPG.0000000000002497

[35] Schindelin J , Arganda-Carreras I, Frise E et al Fiji: an open-source platform for biological-image analysis. Nat Methods 2012;9:676–82. 22743772 10.1038/nmeth.2019 PMC3855844

[36] Kurt-Celep İ , Nihan Kilinc A, Griffin M et al Nitrosylation of tissue transglutaminase enhances fibroblast migration and regulates MMP activation. Matrix Biol 2022;105:1–16. 34763097 10.1016/j.matbio.2021.10.005

[37] Wenthur CJ , Gentry PR, Mathews TP et al Drugs for allosteric sites on receptors. Annu Rev Pharmacol Toxicol 2014;54:165–84. 24111540 10.1146/annurev-pharmtox-010611-134525 PMC4063350

[38] Verma A , Guha S, Diagaradjane P et al Therapeutic significance of elevated tissue transglutaminase expression in pancreatic cancer. Clin Cancer Res 2008;14:2476–83. 18413840 10.1158/1078-0432.CCR-07-4529

[39] Friedrich M , Aigner A. Therapeutic siRNA: state-of-the-art and future perspectives. BioDrugs 2022;36:549–71. 35997897 10.1007/s40259-022-00549-3 PMC9396607

[40] Ahn I , Kang CS, Han J. Where should siRNAs go: applicable organs for siRNA drugs. Exp Mol Med 2023;55:1283–92. 37430086 10.1038/s12276-023-00998-y PMC10393947

[41] Watson WH , Pohl J, Montfort WR et al Redox potential of human thioredoxin 1 and identification of a second dithiol/disulfide motif. J Biol Chem 2003;278:33408–15. 12816947 10.1074/jbc.M211107200

[42] Frickel E-M , Frei P, Bouvier M et al ERp57 is a multifunctional thiol-disulfide oxidoreductase. J Biol Chem 2004;279:18277–87. 14871896 10.1074/jbc.M314089200

[43] Kim S-H , Oh J, Choi J-Y et al Identification of human thioredoxin as a novel IFN-gamma-induced factor: mechanism of induction and its role in cytokine production. BMC Immunol 2008;9:64. 18983687 10.1186/1471-2172-9-64 PMC2596082

[44] Lebreton C , Ménard S, Abed J et al Interactions among secretory immunoglobulin A, CD71, and transglutaminase-2 affect permeability of intestinal epithelial cells to gliadin peptides. Gastroenterology 2012;143:698–707.e4. 22750506 10.1053/j.gastro.2012.05.051

